# Axial growth and refractive change in white European children and young adults: predictive factors for myopia

**DOI:** 10.1038/s41598-020-72240-y

**Published:** 2020-09-16

**Authors:** Sara McCullough, Gary Adamson, Karen M. M. Breslin, Julie F. McClelland, Lesley Doyle, Kathryn J. Saunders

**Affiliations:** 1 Centre for Optometry and Vision Science Research, School of Biomedical Sciences, Ulster University, Coleraine, UK; 2 Psychology Research Institute, School of Psychology, Ulster University, Coleraine, UK

**Keywords:** Health care, Disease prevention, Risk factors, Epidemiology, Paediatric research, Predictive markers

## Abstract

This report describes development of spherical equivalent refraction (SER) and axial length (AL) in two population-based cohorts of white, European children. Predictive factors for myopic growth were explored. Participants were aged 6–7- (n = 390) and 12–13-years (n = 657) at baseline. SER and AL were assessed at baseline and 3, 6 and 9 years prospectively. Between 6 and 16 years: latent growth mixture modelling identified four SER classes (Persistent Emmetropes-PEMM, Persistent Moderate Hyperopes-PMHYP, Persistent High Hyperopes-PHHYP and Emerging Myopes-EMYO) as optimal to characterise refractive progression and two classes to characterise AL. Between 12 and 22-years: five SER classes (PHHYP, PMHYP, PEMM, Low Progressing Myopes-LPMYO and High Progressing Myopes-HPMYO) and four AL classes were identified. EMYO had significantly longer baseline AL (≥ 23.19 mm) (OR 2.5, CI 1.05–5.97) and at least one myopic parent (OR 6.28, CI 1.01–38.93). More myopic SER at 6–7 years (≤ + 0.19D) signalled risk for earlier myopia onset by 10-years in comparison to baseline SER of those who became myopic by 13 or 16 years (p ≤ 0.02). SER and AL progressed more slowly in myopes aged 12–22-years (− 0.16D, 0.15 mm) compared to 6–16-years (− 0.41D, 0.30 mm). These growth trajectories and risk criteria allow prediction of abnormal myopigenic growth and constitute an important resource for developing and testing anti-myopia interventions.

## Introduction

The prevalence of myopia (short-sightedness) is increasing worldwide, reaching epidemic levels in East Asia^1^ where over 90% of 19-year old males in Seoul, South Korea are affected^2^. Recent studies have also highlighted an increasing prevalence in White, European adults^3,4^ and data from our own group (the Northern Ireland Childhood Errors of Refraction [NICER] Study) has shown the number of children with myopia has doubled in the UK over the past 50 years, with children becoming myopic at a younger age than in previous generations^5^. A younger age of onset provides potential for higher levels of myopia to develop throughout childhood as the eye continues to grow.

Although the blurred vision associated with myopic refractive error can be easily treated with spectacles or contact lenses, the myopia problem is more than just optical. Myopia is associated with an increased risk of sight-threatening eye diseases such as retinal detachment, glaucoma and myopic maculopathy and consequently significant visual impairment^6^. Whilst higher levels of myopia confer the greatest risk for associated ocular pathology, even low levels of myopia (− 0.75 to − 3.00D) pose an increased risk of glaucoma, cataract, retinal detachment and myopic maculopathy^7^. Strategies are therefore needed to delay the onset of myopia and slow its progression to avoid visual impairment.

The ability to identify children at risk of myopia, or those who are likely to develop higher levels of myopia, would be advantageous; allowing the opportunity to implement preventative measures such as lifestyle advice (e.g. increasing time spent outdoors, reducing time spent doing near work^8^) or myopia control treatments including pharmacological or optical interventions^9,10^. Population-based prospective data on children’s eye growth and refractive error development using growth curves are rare^11–14^. The Orinda study presents data for predominantly white children in North America^11,14^ and prospective data have been presented for white children living in Sydney, Australia^15^, but there are currently no growth curve data for white, UK children. Ethnicity and geographically specific data are important in respect of understanding typical eye growth as both factors have been shown to significantly impact on myopia prevalence^16,17^.

Growth data are often derived from control arms of myopia treatment trials, reflecting growth of participants who were myopic at the outset of the monitoring period and therefore cannot provide data prior to the onset of myopia which may be predictive. Examining growth patterns can be used to profile refractive risk, provide a platform from which to identify potential myopes prior to onset and stratify emerging myopes according to how fast their myopia is likely to progress.

This prospective, observational study modelled the developmental trajectory of spherical equivalent refractive error (SER) and axial length (AL) in a white population of children and young adults with three main aims. Firstly, previous studies have addressed growth modelling of refractive error and axial length by pre-defining refractive error groups^11,12^. This study modelled SER and AL using latent growth mixture modelling which analyses the data without pre-definitions, to identify clusters of participants who follow similar patterns of growth. Secondly, predictive variables for these homogenous groups were explored. The third aim of the study was to describe percentile growth curves for axial length similar to those used by paediatricians for monitoring paediatric weight and height development^18^ and to determine whether these growth curves could be used to identify children at risk of developing myopia. These types of growth reference charts have recently been used to illustrate eye growth and refractive error change in children by a number of studies of Asian children^13,19,20^ and by Tideman et al^21^ combining data from three European population studies.

## Methods

The NICER Study is a longitudinal study of refractive error development^22^. The study used stratified, random cluster sampling to recruit a representative sample of white children aged 6–7 years (younger cohort) and 12–13 years (older cohort) between 2006 and 2008 (baseline) from schools in Northern Ireland. Data detailing population density and deprivation metrics available from government databases (https://www.nisra.gov.uk/) were used to broadly classify schools into four strata of urban/rural and deprived/not deprived. Stratified random sampling of schools was performed to obtain representation of schools and children across these four strata from four local government districts (Derry, Limavady, Coleraine and Ballymena). The aim was to recruit four primary schools and four post-primary schools from each stratum. All 6–7-year-olds in a primary school and two or more classes of 12–13-year-old children in post-primary schools were invited to participate. A randomisation list was prepared in advance, and where schools were unable or refused to participate, a replacement was identified from the next school on the list within the same stratum.

Participants were assessed prospectively at three, six and nine years (± three months) from baseline.

The study methods have previously been described in detail^23^. In brief, data collection included cycloplegic autorefraction using the binocular open-field autorefractor (either SRW-5000 or NVision-k 5001, Shin-Nippon, Tokyo, Japan). Cycloplegia was induced by one drop of 1.0% cyclopentolate hydrochloride, after corneal anaesthesia with one drop of 0.5% proxymetacaine hydrochloride. Autorefraction was performed at least 20 min after the instillation of cyclopentolate hydrochloride. Confirmation of the absence of the pupillary light reflex and an amplitude of accommodation of less than two dioptres were used to confirm that cycloplegia had been achieved. No less than five readings were taken from which the ‘representative value’ as determined by the instrument was used for further analysis^22^. At least three measurements of axial length, five anterior chamber depth measurements and three corneal curvature measurements were recorded by ocular biometry (IOLMaster, Zeiss, Jena, Germany). A measurement of height was recorded using the Leicester Height Measure (Tanita, UK). After the baseline examination, parents/guardians were asked to complete a questionnaire probing family history of myopia, birth history and the child’s lifestyle.

The study was approved by the University of Ulster’s Research Ethics committee and adhered to the tenets of the Declaration of Helsinki. Written informed consent was obtained from parents or guardians and verbal or written assent was obtained from participants on the day of the examinations.

The autorefraction representative value was used to calculate spherical equivalent refraction (SER) using sphere + cylinder/2. There was a strong correlation between SER and axial length (AL) data from right and left eyes (Spearman’s rho, all *p* < 0.001) therefore only data from right eyes are presented. Myopia was defined as SER of − 0.50 dioptre (D) or less.

### Statistical analysis

Latent growth mixture model was conducted using Mplus v8.2 (Muthén & Muthén, 2018) and was used to identify unobserved growth trajectories of (1) change in SER and (2) AL growth. Model fit was assessed using the Akaike Information Criterion (AIC)^23^, the Bayesian Information Criterion (BIC)^24^ and the sample size-adjusted Bayesian Information Criterion (ssaBIC)^25^. The bootstrap likelihood ratio test (Boot-LRT)^26^ was used to determine when the class-solution became non-significant. Entropy measures were used to determine how accurately participants were classified, with higher values (ranging from 0 to 1) indicating better classification^27^. Graphical interpretation of the class-solution was used concurrently to determine the utility of including an additional class in the model. The ‘best-fit’ was determined by the balance of the fit indices, entropy measures and the number of participants that fell within each class. Missing data were handled using full information maximum likelihood (FIML) which has been found to be an effective method for dealing with missing data in longitudinal designs^28^. Predictive variables for the emergent classes were explored for the younger cohort (odds ratios and confidence intervals, CI) to determine if certain characteristics could be used to predict emerging myopes within this cohort. The 1st, 5th, 10th, 25th, 50th, 75th, 90th, 95th and 99th percentile curves were computed for AL at the four time points (Baseline, three, six and nine years later) for each age cohort. Received operator characteristic (ROC) curves were generated to determine the accuracy of AL percentile curves in determining future myopia for the younger cohort.

Previous studies^29–31^ have analysed changes in refractive error and ocular components according to the age at which myopia onset occurs. For comparison, data within the present study have been similarly analysed to explore differences in the changes demonstrated by participants of the same age who became myopic versus those who remained emmetropic. These data are described per three-year time period due to the wider test interval (three yearly) within the present study compared to other previous studies who conducted annual testing^29–31^. The age of myopia onset is defined as the first time point where a SER of ≤  − 0.50D was manifest given that SER at the previous time point was >  − 0.50D. Those participants with missing data between phases where the age of onset cannot be determined to within the three-year time period are excluded from this analysis. Kruskal–Wallis and Mann–Whitney tests are used to evaluate differences in SER and AL at baseline between those who remained emmetropic and those who became myopic and between myopes by age of myopia onset. Chi-Squared analyses were used to determine associations between parental myopia and age of myopia onset.

## Results

### Participants

Of the 16 primary schools originally identified, 15 participated in the study (94%). Thirteen out of the 15 post-primary schools originally identified participated in the study (87%). Suitable replacement schools were identified and participated in the study. Participation rates were 57% in the younger cohort and 60% in the older cohort. Previously published analysis of the cohort characteristics compared with the underlying population identified that participation rates were not significantly affected by the size of the school or the deprivation or population density of the area in which the school was situated^22^. The ethnicity, sex distribution and type of schooling of the participants were comparable with those of the target population and that of the Northern Irish population as a whole, supporting the assumption that the baseline sample was suitably representative of the underlying population.

Data were collected on a total of 1,047 white children at baseline; 390 of these were aged 6–7 years (younger cohort) and 657 aged 12 − 13 years (older cohort). Data were available from at least two phases from 323 (83%) participants within the younger cohort and for 480 (73%) of the older cohort. Summary data for each study phase on sample size, rate of follow-up, age, gender, ocular biometrics and SER are detailed in Table 1 for the younger and older cohorts.

**Table 1:** Younger cohort aged 6–7 years at baseline, older cohort aged 12–13 years at baseline: Summary data on sample size, time intervals from baseline, rate of follow up, age, gender, ocular biometrics and spherical equivalent refractive error. AL, axial length, CR, corneal radius, AL/CR, axial length to corneal radius ratio, ACD, anterior chamber depth, SER, spherical equivalent refractive error, SD, standard deviation, IQR, inter-quartile range.

Younger cohort				
	Baseline	Phase 2	Phase 3	Phase 4
Time from baseline (years) (IQR)		+ 3.00 (2.97–3.03)	+ 6.10 (5.85–6.16)	+ 9.05 (8.98–9.10)
Sample size	390	295	211	125
Rate of follow up (%)		76	54	32
% male	49.5	46.8	49.3	52.8
Mean age (years) ± SD	7.07 ± 0.38	10.07 ± 0.41	13.08 ± 0.35	16.01 ± 0.36
Median AL (mm) (IQR)	22.56 (22.02–23.07)	23.08 (22.53–23.59)	23.36 (22.82–23.88)	23.65 (23.12–24.26)
Median CR (mm) (IQR)	7.82 (7.63–8.00)	7.82 (7.65–8.02)	7.81 (7.68–8.04)	7.84 (7.70–8.06)
Median AL/CR ratio (IQR)	2.89 (2.83–2.94)	2.95 (2.89–3.01)	2.98 (2.93–3.04)	3.00 (2.93–3.08)
Median ACD (mm) (IQR)	3.45 (3.33–3.59)	3.58 (3.43–3.71)	3.63 (3.49–3.78)	3.70 (3.52–3.85)
SER (D) (IQR)	+ 1.13 (+ 0.63 to + 1.75)	+ 0.75 (+ 0.25 to + 1.25)	+ 0.75 (+ 0.25 to + 1.25)	+ 0.68 (-0.25 to + 1.13)

Older cohort				
	Baseline	Phase 2	Phase 3	Phase 4
Time from baseline (years) (IQR)		+ 2.97 (2.88–2.99)	+ 6.04 (+ 5.81–6.18)	+ 8.92 (8.86–9.00)
Sample size	657	427	225	113
Rate of follow up (%)		65	34	17
% male	50.5	45.0	43.1	36.3
Mean age (years) ± SD	13.09 ± 0.36	16.05 ± 0.32	19.20 ± 0.42	21.98 ± 0.35
Median AL (mm) (IQR)	23.30 (22.71–23.91)	23.44 (22.84–24.09)	23.47 (22.84–24.19)	23.69 (23.04–24.54)
Median CR (mm) (IQR)	7.92 (7.73–8.08)	7.93 (7.75–8.09)	7.90 (7.74–8.08)	7.98 (7.73–8.14)
Median AL/CR ratio (IQR)	2.97 (2.92–3.03)	2.98 (2.93–3.05)	3.00 (2.93–3.05)	3.01 (2.94–3.07)
Median ACD (mm) (IQR)	3.62 (3.47–3.78)	3.67 (3.48–3.84)	3.65 (3.51–3.83)	3.68 (3.51–3.88)
SER (D) (IQR)	+ 0.50 (+ 0.00 to + 1.13)	+ 0.50 (-0.13 to + 1.13)	+ 0.75 (-0.13 to + 1.50)	+ 0.69 (-0.25 to + 1.38)

There was no statistically significant difference in baseline refractive error and socio-economic indicators (socio-economic rank and parental education) between participants and non-participants in either the younger or older cohorts at phase 2, 3 or 4. Females in the older cohort were statistically significantly more likely to participate than males at Phases 2, 3 and 4 (p ≤ 0.005). Those in the younger cohort who had at least one myopic parent were statistically significantly more likely to participate at Phase 3 compared to those with no myopic parents (χ^2^ = 10.51, p = 0.001). However, there was no statistically significant difference in parental myopia in the younger cohort at phase 2 or 4 (p ≥ 0.15) or for the older cohort at phase 2, 3 or 4 (p ≥ 0.17). Spectacle wearers in the older cohort were statistically significantly more likely to participate than non-spectacle wearers at phase 3 (χ^2^ = 5.45, p = 0.02). There was no statistically significant difference in spectacle wear in participants and non-participants in the younger cohort at phase 2, 3 or 4 (p ≥ 0.09), or in the older cohort at phase 2 or 4 (p ≥ 0.10).

### Latent growth mixture modelling and predictive variables

**Figure 1:**
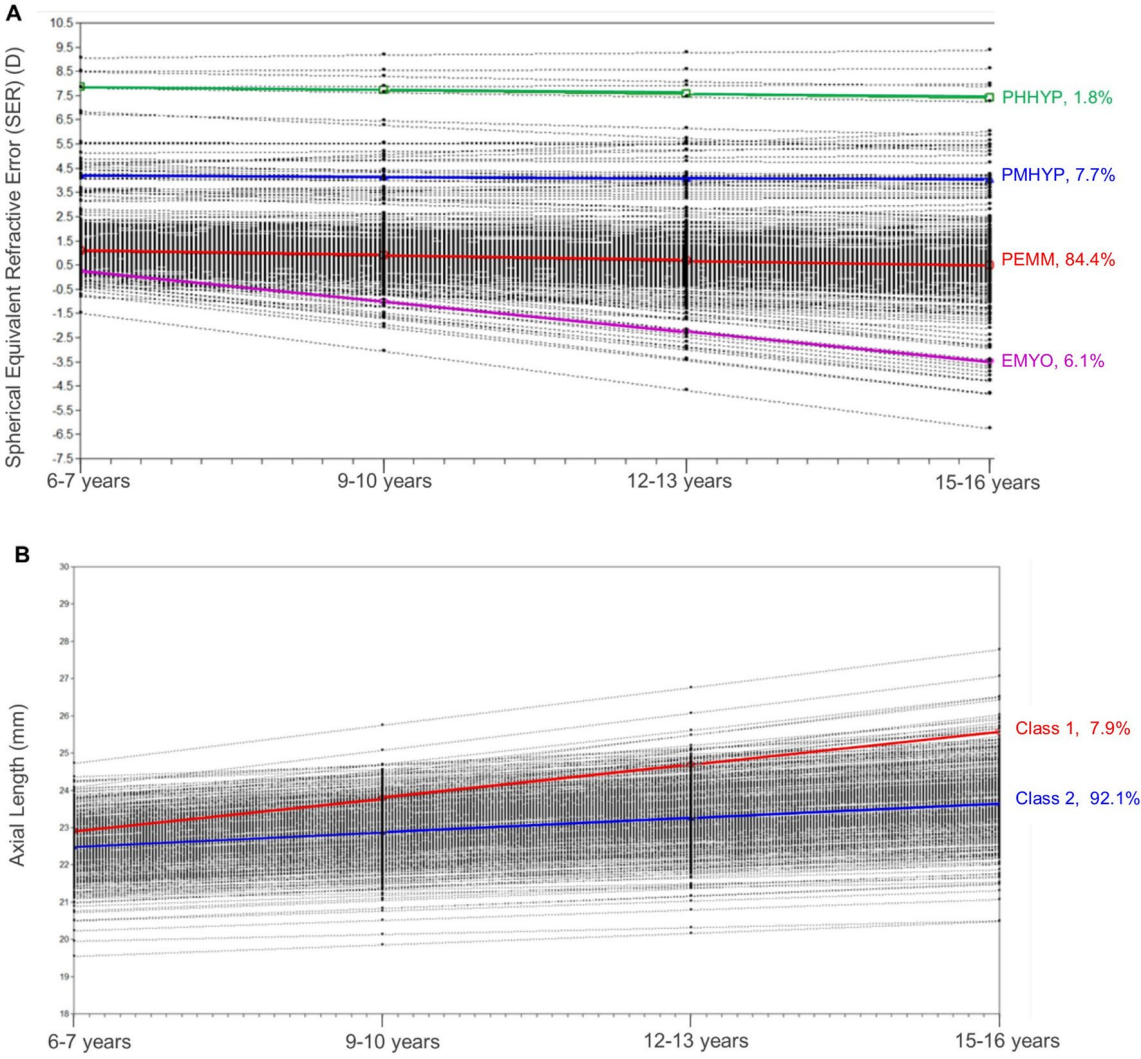
(**A**) Spherical equivalent refractive error (SER) and (**B**) Axial Length (AL) growth models for the younger cohort between 6–7 and 15–16 years. SER classes are labelled as PHHYP = persistent high hyperopes, PMHYP = persistent moderate hyperopes, PEMM = persistent emmetropes, EMYO = emerging myopes. Black dots and lines represent individual participant data.

Latent growth mixture modelling showed a four-class solution was the best fit for SER and a two-class solution for AL for the younger cohort (the fit indices are detailed in Supplementary Materials 1 and 2). The resultant four refractive error classes are labelled as ‘Persistent Emmetropes-PEMM’ (n = 329, 84.4%), ‘Persistent Moderate Hyperopes- PMHYP’ (n = 30, 7.7%), ‘Persistent High Hyperopes-PHHYP’ (n = 7, 1.8%) and ‘Emerging Myopes-EMYO’ (n = 24, 6.1%) and the two classes for axial length are labelled as ‘Class 1’ (n = 31, 7.9%) and ‘Class 2’ (n = 359, 92.1%). Figure 1A,B show the graphical presentation of the growth model of SER and AL respectively for the younger cohort between 6–7 and 15–16 years and the percentage of participants within each class.

**Figure 2:**
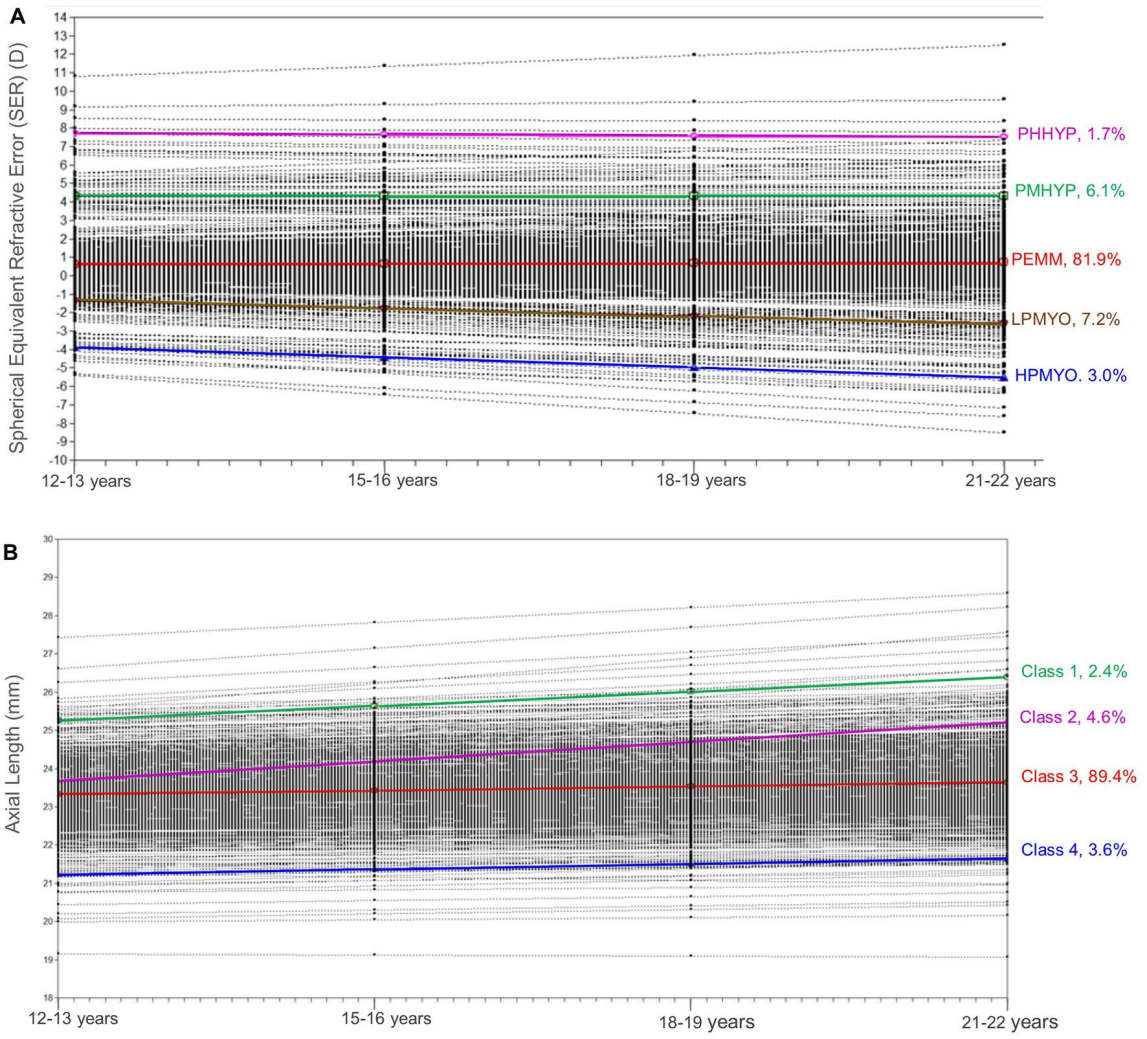
(**A**) Spherical Equivalent Refractive Error (SER) and (**B**) Axial Length (AL) growth models for the older cohort between 12–22 years. SER classes are labelled as PHHYP = persistent high hyperopes, PMHYP = persistent moderate hyperopes, PEMM = persistent emmetropes, LPMYO = low progressing myopes and HPMYO = high progressing myopes. Black dots and lines represent individual participant data.

A five-class solution provided the best fit for SER and a four-class solution for AL for the older cohort (the fit indices for SER and AL for the older cohort are detailed in Supplementary Materials 3 and 4). The emerging five refractive error classes are labelled as ‘Persistent Emmetropes-PEMM’ (n = 538, 81.9%), ‘Persistent Moderate Hyperopes- PMHYP’ (n = 40, 6.1%), ‘Persistent High Hyperopes-PHHYP’ (n = 11, 1.7%), Low Progressing Myopes-LPMYO’ (n = 48, 7.2%) and High Progressing Myopes-HPMYO’ (n = 20, 3.0%)^32^ and axial length classes as ‘Class 1’ (n = 16, 2.4%), ‘Class 2’ (n = 30, 4.6%), ‘Class 3’ (n = 587, 89.4%) and ‘Class 4’ (n = 24, 3.6%). Figure 2A,B show the graphical presentation of the growth model of SER and AL respectively for the older cohort aged between 12–13 and 12–22 years and the percentage of participants within each class. Table 2 shows the initial status, which is the average starting position for the class for either SER or AL and the slope of the line, which indicates the average change in SER or AL per three-year time period for each class for the younger cohort and older cohorts.

**Table 2:** Latent growth mixture modelling results: the initial status (average class starting point for SER and AL) and slope (average change in SER and AL over each three year period) for the younger cohort and older cohorts.

Younger cohort		
Class	Initial status	Slope
	SER (D)	SER (D)
Persistent high hyperopes	+ 7.856	− 0.153
Persistent moderate hyperopes	+ 4.193	− 0.053
Persistent emmetropes	+ 1.068	− 0.197
Emerging myopes	+ 0.209	− 1.230

	AL (mm)	AL (mm)
1	22.901	0.889
2	22.483	0.386

Older cohort		
Class	Initial status	Slope
	SER (D)	SER (D)
Persistent high hyperopes	+ 7.722	− 0.079
Persistent moderate hyperopes	+ 4.281	+ 0.011
Persistent emmetropes	+ 0.579	+ 0.046
Low progressing myopes	− 1.364	− 0.406
High progressing myopes	− 3.884	− 0.545

	AL (mm)	AL (mm)
1	25.253	0.377
2	23.663	0.518
3	23.316	0.104
4	21.253	0.130

Table 3 details the logistic regression analysis (Odds ratios [OR] and 95% Confidence Intervals [CI]) on the predictive variables associated with being classed within the ‘Emerging Myopes-EMYO’ class compared to the ‘Persistent Emmetropes-PEMM’ class within the younger cohort.

**Table 3:** Logistic regression predictive analysis for ‘Emerging Myopes-EMYO’ compared to ‘Persistent Emmetropes-PEMM’ for the younger cohort (between 6–7 and 15–16 years). Statistically significant predictive variables are underlined and in italics.

Predictive variables for emerging myopes	Odds ratio (95% confidence interval)
**Gender** Male 1, female 0	0.37 (0.11 to 1.23) p = 0.105
**Socioeconomic status** Quintiles 1 to 5, 1 = low, 5 = high	1.18 (0.81 to 1.74) p = 0.393
***At least one parent myopic*** *yes 1, no 0*	*6.28 (1.01 to 38.93) p = 0.048*
**Physical activity** Sedentary 1, light activity 2, regular activity up to 3 h/week 3, regular activity > 3 h/week 4	0.47 (0.19 to 1.19) p = 0.113
**Time spent doing nearwork** Average hrs/week	0.76 (0.36 to 1.61) p = 0.471
**BMI** Per unit increase	1.00 (0.74 to 1.36) p = 0.982
**Height** Per m increase	0.14 (0 to 335.91) p = 0.625
**Breastfed** Yes 1, no 0	1.03 (0.47 to 2.28) p = 0.933
***Axial length at baseline*** *Per mm increase*	*2.50 (1.05 to 5.87) p = 0.038*

### Percentile growth curves

**Figure 3:**
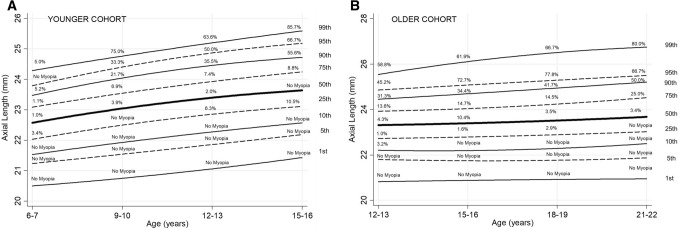
(**A**) Percentile curves (1st to 99th) of AL for the younger cohort aged between 6–7 years and 15–16 years. Percentages = number of participants classed as myopic/number of participants within the percentile × 100%. (**B)** Percentile curves (1st to 99th) of AL for the older cohort aged between 12–13 years and 21–22 years. Percentages = number of participants classed as myopic/number of participants within the percentile × 100%.

As AL at baseline was significantly predictive of those likely to be grouped within the ‘Emerging Myopes’ class for the younger cohort, percentile growth curves of AL were explored to determine if they could be clinically useful in the prediction of those likely to become myopic. Figure 3A,B shows the percentile curves for AL for the younger and older cohorts respectively. Also detailed are the percentage of participants falling within each percentile who were myopic at each time point. Table 4 details the percentile data of AL by age for the younger and older cohorts. All percentiles showed growth in AL between 6–7 and 15–16 years with those percentiles above the 5th growing more than 1 mm. The 95th centile showed the greatest change in AL from 23.76 mm at 6–7 years to 25.21 mm at 15–16 years (change in AL of 1.45 mm). The percentage of those classed as myopic increased with increasing percentile with the greatest risk of myopia occurring in the 90th centiles and above. There were no participants classed as myopic who fell below the 25th percentile.

**Table 4:** Centile values of axial length (mm) for the younger (6–7 to 15–16 years) and older (12–13 to 21–22 years) cohorts (1st–99th).

Age (years)	Younger cohort								
	Percentiles								
	1st	5th	10th	25th	50th	75th	90th	95th	99th
6–7	20.49	21.22	21.52	22.02	22.56	23.07	23.46	23.76	24.25
9–10	20.78	21.59	21.96	22.53	23.08	23.59	24.08	24.49	24.88
12–13	21.03	21.81	22.24	22.82	23.36	23.88	24.42	24.78	25.08
15–16	21.44	22.20	22.58	23.12	23.65	24.26	24.73	25.21	25.63
9 year change	0.95	0.98	1.06	1.10	1.09	1.19	1.27	1.45	1.38
Approx. annual change	0.11	0.11	0.12	0.12	0.12	0.13	0.14	0.16	0.15

Age (years)	Older cohort								
	Percentiles								
	1st	5th	10th	25th	50th	75th	90th	95th	99th
12–13	20.79	21.76	22.17	22.71	23.30	23.91	24.46	24.86	25.54
15–16	20.96	21.86	22.3	22.84	23.44	24.09	24.67	25.08	26.12
18–19	20.83	21.64	22.18	22.84	23.47	24.19	24.97	25.29	26.5
21–22	20.97	21.91	22.53	23.04	23.69	24.54	25.20	25.50	26.75
9 year change	0.18	0.15	0.36	0.33	0.39	0.63	0.74	0.64	1.21
Approx. annual change	0.02	0.02	0.04	0.04	0.04	0.07	0.08	0.07	0.13

Between 12–13 and 21–22 years there was little change in AL percentiles on or below the median, with less than 0.5 mm change. The 99th centile showed the greatest change over time increasing from 25.54 to 26.75 mm (1.21 mm change in AL) between baseline and Phase 4. Similar to the younger cohort, the percentage of those classed as myopic increased with increasing percentile with the greatest risk of myopia occurring in the 90th centiles and above. There were very few participants classed as myopic who fell on or below the median centile.

Receiver Operator Characteristics (ROC) Curve analysis was used to determine the best SER and AL percentile cut-off to identify those participants within the younger cohort who were not myopic aged 6–7 years but became myopic at Phase 2, 3 or 4. The best balance of sensitivity and specificity for AL occurred with a cut-off of 23.07 mm (greater than the 75th centile (sensitivity 48.89%, specificity 80.37%, area under the curve = 0.6904). A cut-off of greater than 22.56 mm (greater than the 50th centile) improved sensitivity to 68.89% but specificity reduced to 55.19%. To improve the predictive ability of the growth charts, an additional criterion of moving up at least one centile of AL growth between 6–7 and subsequent Phases was interrogated alongside an AL ≥ 23.07 mm at 6–7 years. This is similar to the interpretation of growth charts for children of height and weight, where a growth (or delay in growth) across one centile is used to indicate a growth anomaly^18^. This enhanced criteria correctly identified 89% (sensitivity) of participants who became myopic at subsequent Phases (n = 40/45); 5 participants (11%) who became myopic showed neither of these AL characteristics. Conversely, 31% (n = 83/270) of those who did not become myopic by age 15–16 years also demonstrated at least one of these characteristics of AL (specificity = 69.26%).

ROC curve analysis was also used to determine the predictive value of SER at baseline in order to identify those participants within the younger cohort who were not myopic aged 6–7 years but became myopic by Phase 2, 3 or 4. The best balance of sensitivity and specificity occurred with a cut-off of SER less than + 0.63D (75th centile [sensitivity 75.56%, specificity 82.96%, area under the curve = 0.8698]). A cut-off of less than + 1.13D (50th centile) improved sensitivity to 97.78% but specificity reduced to 55.56%. Combining a cut-off of SER less than + 0.63D and a cut-off of AL larger than 23.07 mm or moving up at least one centile of AL growth between 6 and 7 and subsequent Phases resulted in sensitivity and specificity of 64.44% and 95.40% respectively.

Within both cohorts there were a number of participants with axial lengths classified as greater than the 75th centiles (> 24.26 mm younger, n = 11; > 24.54 mm older, n = 12) who were not myopic at the last data collection point (either 15–16 years or 21–22 years). These participants were found to have flatter than average corneas than their peers (median younger = 8.12 mm, range 7.86–8.45 mm; median older = 8.22 mm, range 7.89–8.82 mm) and all these participants within both cohorts, except one, had axial lengths greater than the 75th centile at their first visit and all subsequent visits. The participants were all taller than average, with heights consistently above the 90th centile and above the 75th centile in the younger and older cohorts respectively^18^.

### Change in SER and AL by age of myopia onset

**Figure 4:**
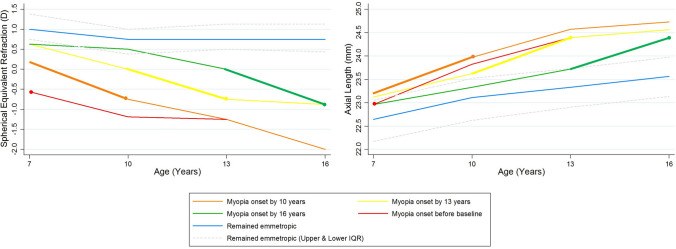
Median SER (left) and AL (right) data by age separated into age of myopia onset subgroups. Median and IQR data for those who remained emmetropic are also plotted. The dots indicate the time point when the group became myopic. Pre-myopic slopes are indicated by a bold line.

Figure 4 shows the median change in SER and AL grouped by the age of onset of myopia for the younger cohort. Median and IQR data are presented for those participants who remained emmetropic. Data have not been presented for the older cohort due to the small number of data points available for participants becoming myopic after 16 years of age (n = 4). The rate of change in both SER and AL estimated in the three years prior to the onset of myopia is similar, regardless of the age of onset. On average this was a myopic shift of approximately − 0.85D and an increase in axial length of 0.74 mm axial growth in the three-year period prior to myopia being identified. Kruskal–Wallis analyses showed a statistically significant difference in SER (χ^2^ = 47.72, p = 0.0001) at baseline for those who remained emmetropic (n = 196, median =  + 1.00D, IQR + 0.75 to + 1.38D) compared to those who became myopic at any age during the monitoring period (n = 40, median =  + 0.31D, IQR + 0.06 to + 0.69D) (Mann–Whitney, pairwise comparisons, all p < 0.015). Additionally, those who became myopic by aged 10 years had a significantly lower median SER (n = 22, + 0.19D, IQR − 0.13 to + 0.38D) at baseline (6–7 years) compared to those who became myopic by age 13 years (n = 11, median =  + 0.63D, IQR + 0.38 to + 1.00D; z =  − 3.29, p = 0.001) and by age 16 years (n = 7, median =  + 0.63D, IQR + 0.25 to + 0.88D; z =  − 2.44, p = 0.02). A statistically significant difference in AL was also found for those who remained emmetropic compared to those who became myopic (χ^2^ = 13.57, p = 0.0036); however, pairwise comparisons showed the difference to be significant only between those who remained emmetropic and those who became myopic by 10 years (Mann–Whitney z =  − 3.424, p = 0.0006). Although AL was longer on average at baseline for those who became myopic by 10 years (median = 23.19 mm, IQR 22.76 to 23.43 mm) compared to by 13 (median = 23.12 mm, IQR 22.29 to 23.48 mm) and 16 years (median = 22.96 mm, IQR 22.58 to 23.00 mm), this was not statistically significant (10 vs 13 years z =  − 0.78, p = 0.44; 10 vs 16 z = 1.33, p = 0.18). Myopia onset was linked to a similar AL value (median 24.12 mm; IQR 23.57 to 24.28 mm), regardless of age of onset (χ^2^ = 2.53, p = 0.282). Those who became myopic by age 10 and 13 years were significantly more likely to have at least one myopic parent compared to those who became myopic by 16 years of age (10 vs 16 years, χ^2^ = 9.63, p = 0.002; 13 vs 16 years, χ^2^ = 5.00, p = 0.025). Table 5 summarises the characteristics for myopia development by age of onset.

**Table 5:** Characteristic features of SER, AL and parental history of myopia by age of onset of myopia.

Age of myopia onset	SER at 6–7 years Median (IQR) (D)	Axial length at 6–7 Median (IQR) (mm)	At least one myopic parent?
By 10 years old	0.19 − 0.13 to 0.38	23.19 22.76 to 23.43	Yes
By 13 years old	0.63 0.38 to 1.00	23.12 22.29 to 23.48	Yes
By 16 years old	0.63 0.25 to 0.88	22.96 22.58 to 23.00	No

## Discussion

This study provides novel population-specific prospective data for SER change and AL growth in white children living in the UK; a valuable resource for researchers, eye care practitioners and those developing and testing anti-myopia interventions. Four distinct classes of refractive development were evident from childhood to teenage years and five distinct classes from teenage years to adulthood. Jones et al.^11^. (Orinda Longitudinal Study of Myopia) used four refractive groups (Myopes, Emmetropising Hyperopes, Emmetropes and Persistent Hyperopes) to compare ocular component growth curves in their study of children 6–15 years. These categories differ to those derived through recognised growth modelling techniques used in the present study. Our analyses detected four classes of refractive development within the younger cohort; Emerging Myopes, Persistent High Hyperopes, Persistent Moderate Hyperopes and Persistent Emmetropes.

Notwithstanding the differences in approach to categorisation, the Persistent Emmetropes in the present study had a similar baseline (approximately + 1.00D) and rate of change in SER (approximately − 0.20D per three-year time period) to that reported by Jones et al.^11^. Wong et al.^12^ identified similar refractive groups to the present study when examining Chinese children aged 7–12 years in Singapore. The Singaporean data revealed a persistent myopic group, which was not apparent in UK white children at 6–7 years. It is worth noting that whilst Wong and Jones et al. both identified a persistent hyperope group in their cohorts, the magnitude of hyperopia amongst persistent hyperopes reported in Singapore and the United States is markedly lower than that of persistent hyperopes in the present study.

The latent growth mixture model generated an average baseline refractive error of + 0.21D at 6–7 years with an average change of − 1.23D over a three-year period (approximately − 0.41D annually). The addition of a fifth refractive class in the latent growth mixture model of the younger cohort data set did not differentiate emerging myopes into fast/slow progressors, rather the additional class further distilled the hyperopes in relation to their baseline SER.

In agreement with data from Singaporean children aged eight to 13 years^31^, white UK children who became myopic between six and 16 years of age demonstrated an accelerated shift in SER and AL in the three-year period preceding myopia onset (Fig. 4). Once myopia was established, our results suggest that a younger age of onset is associated with more rapid rate of progression, in agreement with reports from the COMET (Correction of Myopia Evaluation Trial) and SCORM (Singapore Cohort Of the Risk factors for Myopia) cohorts^33,34^. The notion that later onset age is related to slower progression once myopia is established was further apparent in the current study when comparing myopic progression and AL elongation between the younger and older cohorts. The rate of progression of myopia and AL elongation was approximately 2–3 times slower between 12 and 22 years in the older cohort compared to that seen between 6 and 16 years. Myopia in our cohort of white UK children appears to progress more slowly than that described by Rozema et al.^31^ for Singaporean children living in Singapore. Rozema et al. report an average increase in myopia of − 1.04D over the three-year period following myopia onset at 10 years of age. In the NICER cohort, myopia increased on average by − 0.50D over the three-year period following myopia onset at 10 years.

The five-class solution for SER change between 12 and 22 years showed an absence of an ‘Emerging Myopes’ class reflecting the low incidence of myopia during this timeframe. Axial elongation continues during teenage years and into adulthood and future follow-up of this cohort would be helpful to explore when myopia progression and axial growth ceases. Further long-term evaluation may also detect individuals who develop adult-onset myopia after 22 years of age, a phenomenon which has been reported by other studies^21,35^.

The two-class solution for axial growth in children between 6 and 16 years (compared to the four-class for SER) illustrates that ocular components, such as crystalline lens power, shape and thickness, are important determinants of SER alongside AL during this period of development. Average corneal radius remained relatively constant between 6 and 16 years suggesting that compensatory changes in the crystalline lens parameters and deepening of the anterior chamber oppose the increase in AL seen during this period. Mutti et al.^30^, from the Collaborative Longitudinal Evaluation of Ethnicity and Refractive Error Study report a flattening and thinning of the crystalline lens to compensate for the typical axial elongation recorded during early childhood. In agreement with the recent report from Hagen et al.^36^ who assessed longitudinal eye growth in Norwegian adolescents between 16 to 18 years of age, our results demonstrate that the eye continues to grow between 16 and 22 years, although at very slow rate (approximately 0.03–0.04 mm per year), in those who showed persistent emmetropia and hyperopia. This suggests that small compensatory changes in lens thickness and curvature may occur to promote the maintenance of emmetropia during this late teenage period.

Estimated average annual changes in axial length were small, approximately 0.3 mm in those with accelerated growth in the younger cohort compared to 0.13 mm among those demonstrating more consistent eye growth. This highlights the need for instrumentation that can precisely measure and monitor eye growth when myopia control is being practised.

Parental history of myopia and longer AL at 6–7 years are risk factors for emergent myopia in childhood. Children with at least one myopic parent were over six times more likely to be classified within the Emerging Myopes refractive group compared to the Persistent Emmetropes group (OR = 6.28 [95% CI 1.01 to 38.93]). Meta-analysis by Zhang et al.^37^ report a significant positive association between parental myopia and a child’s risk of developing myopia, however they report a lower odds ratio of 1.53 (95% CI 1.21–1.85) when one parent had myopia and 2.19 (95% CI 1.42–2.77) when two parents were myopic. Individual studies within this meta-analysis have a range of odds ratios for risk of myopia development from 1.48 to 7.90. While the current study’s odd ratios fall at the higher end of this range, potentially due to cohort specific differences, Zhang et al.’s data were polled from different populations, and across a variety of ages and ethnicities.

Within the present study, participants from both cohorts who had an axial length that fell on or above the 90th centile of growth were at the greatest risk of myopia. The most predictive AL centile for the younger cohort for future myopia was greater than 23.07 mm at 6–7 years, however, while specificity (80.37%) was high, the sensitivity (48.89%) was relatively poor. In paediatric medicine, the interpretation of growth charts includes monitoring whether a child crosses one centile space as this may indicate a growth anomaly^18^. Using a combination of two criterion, either (1) the child had an AL > 23.07 mm at 6–7 years or (2) showed an increase in at least one centile of growth improved the sensitivity (89%) whilst maintaining a reasonable specificity (69%) for future myopia. No child with an axial length below the 25th centile of growth between 6 and 16 years was myopic and very few participants within the older cohort whose AL fell below the median centile were myopic. Given that measurement of axial length is not routinely accessible in current general eye care practice, it is useful to note that, in line with previous reports, a SER of + 0.63DS or less at 6–7 years is relatively sensitive (75.56%) and specific (82.96%) in predicting myopia development in our population^38^. However, the growth charts and risk criterion for myopia development presented here can be used by researchers and clinicians wishing to detect excessive eye growth at an early age and incorporated into clinical advice and management plans discussed with patients and parents.

Tideman et al.^21^ also presented percentile growth charts for AL using data compiled from three separate European studies of children and adults. The 50th and 95th centiles for the youngest children (6–7 years) are comparable with the present study. The 50th centile for adults within the Tideman report (> 45 years) are comparable to the adult data within the present study (21–22 years), however, the 95th centile reported by Tideman is longer (26.18 mm) than that found in the present study (25.50 mm). This disparity may be explained by the difference in age between the two populations or the higher magnitudes and greater prevalence of high myopia (maximum SER − 9.8D) present in the Tideman data compared to the present study (maximum SER − 8.75D, n = 2 high myopes). In contrast to the present study, Tideman reports almost all adult participants with AL greater than or equal to the 90th centile (approximately greater than 25 mm) in the Rotterdam cohort were myopic. We found 27% within both the younger and older cohorts respectively of those with an AL of greater than or equal to the 90th centile (AL > 24.73 mm at 16 years; AL > 25.24 mm at 22 years) were not myopic. These participants were found to have flatter than average corneas and were likely to have longer eyes (≥ 90th centile) across the monitoring period. They were also found to be taller than average. These participants may develop adult onset myopia and with longer monitoring periods our data may look similar to that of Tideman once these participants reach 45 years old. However, it is interesting to consider whether these non-myopic individuals with long axial lengths are at comparable risk of future ‘myopic’ pathology as those with axial myopia, or does a consistently large eye with a regular growth pattern have a better long-term outcome than one which demonstrates acceleration in growth and crosses the centiles? Characterisation of retinal nerve fibre thickness in myopic compared with non-myopic eyes of similar axial length and long-term follow-up of these participants would be helpful to explore this further.

There are notable differences in the refractive error distribution and axial length data within the present study compared to those studies presenting similar data from children and adults of East Asian descent. Diez et al.^13^ present percentile growth curves of axial length for Chinese schoolchildren. Median centiles are comparable or in fact slightly shorter for their children at age six years compared to the equivalent age group of the present study; however, by age nine and 15 years, there is a disparity of 0.64 mm and 0.77 mm in AL for the median centiles respectively indicating marked accelerated eye growth in children of East Asian descent compared to white UK children. These differences reinforce the need for population and geographic specific normative data.

All participants within the younger cohort who became myopic by at least 16 years of age had a significantly lower SER at 6–7 years compared to those who remained emmetropic. Those participants who became myopic by the age of ten years also had a significantly lower SER at 6–7 years compared to those who became myopic by 13 and 16 years. Participants who became myopic by ten and 13 years were significantly more likely to have at least one myopic parent compared to those who became myopic by 16 years of age. While AL at baseline as a single parameter, was less helpful in predicting future myopia, those who became myopic by ten years had a significantly longer AL at 6–7 years compared to those who remained emmetropic. Using these variables in combination, clinicians should appreciate that children presenting at 6–7 years with low SER (≤ + 0.19D), with at least one myopic parent and longer axial lengths (≥ 23.19 mm) are likely to develop myopia by age 10 years. In such cases, it would be helpful to advise parents and children of environmental and lifestyle modifications (e.g. increasing time spent outdoors, reducing time spent doing near work^8^) that may delay the onset of myopia and consequently result in lower magnitudes of myopia in adult life. Clinicians may also wish to instigate careful monitoring to allow early application of interventions when myopia manifests.

Children presenting with slightly more hyperopic SER at 6–7 years (< + 0.63D) with at least one myopic parent are likely to develop myopia by age 13 years and those with the same SER at 6–7 years with no myopic parents are likely to develop myopia by 16 years. Children with a SER of ≥ + 1.00D at 6–7 years are unlikely to develop myopia. Conversely, where clinicians notice a rapid change in SER or AL myopia onset can be anticipated within at least three years.

Attrition in participation occurred over the nine years of the present study. However, this attrition had relatively little impact on the profile of the participants; the characteristics of participants in 3, 6 and 9-year follow-up testing was similar to those who didn’t participate at follow-up, supporting the assumption that the data and outputs from the present analyses can be considered representative of the baseline cohort and the underlying population. While older cohort participants were statistically significantly more likely to participate than non-spectacle wearers at 6-year follow-up, this difference wasn’t evident for those participating at 3 or 9-years after baseline and the range of refractive errors worn by participants was extensive, ranging from − 7.00D to + 9.00D.

Whilst it is now well established that increased time spent outdoors during childhood is protective against the onset of myopia^8^, baseline data collection for the NICER study commenced in 2006 and time spent outdoors was not a metric included in data collection. As a result, the present analysis was unable to include time spent outdoors as a factor in relation to the emergence of myopia or the maintenance of emmetropia.

Growth patterns relating to high myopia (≤ − 6.00D) could not be considered in the present study as very few participants (n = 2) within this population met this criterion.

This is the first study to present prospective change in cycloplegic SER and AL growth through childhood and early adult years for a large cohort of white, European children and young adults. The present study used cycloplegic measures of refractive error (1.0% cyclopentolate hydrochloride) when determining ocular parameters associated with risk of future myopia. Hence, clinicians wishing to use SER values to advise parents on a child’s likelihood of future myopia need to use cycloplegic methods to determine SER because non-cycloplegic autorefraction or retinoscopy outcomes are known to overestimate the presence and magnitude of myopia in childhood^39,40^.

## Conclusions

These novel prospective population-specific growth trajectories for SER and axial growth in white children and young adults living in the UK are a valuable resource for researchers, eye care practitioners and those developing and testing anti-myopia intervention. Children with lower SER (≤ 0.19D), at least one myopic parent and longer axial lengths (≥ 23.19 mm) at age 6–7 years were at greatest risk of developing myopia by ten years of age. Children with AL growth profiles that crossed one centile were also more likely to develop myopia by 16 years of age than those with more consistent growth patterns. The profile of axial growth may be more important indicator of future visual impairment than the absolute magnitude of AL. Eye growth was considerably slower, on average, between 12 and 22 years than between 6 and 16 years demonstrating that delaying the onset of myopia is a key consideration in constraining the magnitude of myopic outcomes.

## Supplementary information

Supplementary information.

## Data Availability

The datasets generated and analysed during the current study are not publicly available to protect potentially identifiable information on the human participants involved but are available from the corresponding author on reasonable request.
