# Supplementary material for: Axial growth and refractive change in white European children and young adults: predictive factors for myopia
Source: Sci Rep. 2020 Sep 16;10:15189. doi: 10.1038/s41598-020-72240-y (PMC7494927; doi:10.1038/s41598-020-72240-y)
Supplement: Supplementary file 1 — Supplementary information. [file 41598_2020_72240_MOESM1_ESM.docx]

**Title:** Axial Growth and Refractive Change in White European Children and Young Adults: Predictive Factors for Myopia

**Authors:** Sara McCullough^1^, Gary Adamson^2^, Karen MM Breslin^1^, Julie F McClelland^1^, Lesley Doyle^1^, Kathryn Saunders*^1^

1 Centre for Optometry & Vision Science Research, School of Biomedical Sciences, Ulster University

2 Psychology Research Institute, School of Psychology, Ulster University

**Supplementary Material**

| **Model** | **Log Likelihood** | **#parameters** | **AIC** | **BIC** | **ssaBIC** | **Boot-LRT (*p*)** | **Entropy** |
| --- | --- | --- | --- | --- | --- | --- | --- |
| **1-class** | -1293.508 | 9 | 2605.016 | 2640.711 | 2612.155 | NA | NA |
| **2-class** | -1205.534 | 12 | 2435.068 | 2482.661 | 2444.586 | 0.0000 | 0.970 |
| **3-class** | -1175.339 | 15 | 2380.679 | 2440.171 | 2392.577 | 0.0000 | 0.971 |
| ***4-class*** | *-1146.329* | *18* | *2328.657* | *2400.048* | *2342.935* | *0.0000* | *0.929* |
| **5-class** | -1139.631 | 21 | 2321.262 | 2404.551 | 2337.919 | 0.0400 | 0.928 |

**Supplementary Material 1.** Details of the fit indices of the latent growth mixture modelling of Spherical Equivalent Refractive Error (SER) for the younger cohort, aged 6-16 years. AIC=Akaike Information Criterion, BIC= Bayesian Information Criterion, ssaBIC= sample size-adjusted Bayesian Information Criterion, Boot-LRT= Bootstrap Likelihood Ratio Test. The best fit for this parameter for this cohort is underlined and in italics.

| **Model** | **Log Likelihood** | **#parameters** | **AIC** | **BIC** | **SSABIC** | **Boot-LRT (*p*)** | **Entropy** |
| --- | --- | --- | --- | --- | --- | --- | --- |
| **1-class** | -496.981 | 9 | 1011.961 | 1047.657 | 1019.100 | NA | NA |
| ***2-class*** | *-458.206* | *12* | *940.412* | *988.005* | *949.930* | *0.0000* | *0.861* |
| **3-class** | -454.521 | 15 | 939.042 | 998.535 | 950.941 | 0.1579 | 0.835 |

**Supplementary Material 2.** Details of the fit indices of the latent growth mixture modelling of Axial Length (AL) for the younger cohort, aged 6-16 years. AIC=Akaike Information Criterion, BIC= Bayesian Information Criterion, ssaBIC= sample size-adjusted Bayesian Information Criterion, Boot-LRT= Bootstrap Likelihood Ratio Test. The best fit for this parameter for this cohort is underlined and in italics.

| **Model** | **Log Likelihood** | **#parameters** | **AIC** | **BIC** | **SSABIC** | **Boot-LRT (*p*)** | **Entropy** |
| --- | --- | --- | --- | --- | --- | --- | --- |
| **1-class** | -1853.387 | 9 | 3724.774 | 3765.163 | 3736.588 | NA | NA |
| **2-class** | -1752.642 | 12 | 3529.283 | 3583.136 | 3545.035 | 0.0000 | 0.969 |
| **3-class** | -1688.135 | 15 | 3406.270 | 3473.585 | 3425.960 | 0.0000 | 0.967 |
| **4-class** | -1653.567 | 18 | 3343.133 | 3423.912 | 3366.761 | 0.0000 | 0.970 |
| **5-class** | -1639.607 | 21 | 3321.213 | 3415.455 | 3348.779 | 0.0000 | 0.935 |
| **6-class** | -1626.298 | 24 | 3300.596 | 3408.301 | 3332.100 | 0.0000 | 0.937 |

**Supplementary Material 3**. Details of the fit indices of the latent growth mixture modelling of Axial Length (AL) for the older cohort, aged 12-22 years. AIC=Akaike Information Criterion, BIC= Bayesian Information Criterion, ssaBIC= sample size-adjusted Bayesian Information Criterion, Boot-LRT= Bootstrap Likelihood Ratio Test. The grey shaded section indicates the best fit for this parameter for this cohort.

| **Model** | **Log Likelihood** | **#parameters** | **AIC** | **BIC** | **SSABIC** | **Boot-LRT (*p*)** | **Entropy** |
| --- | --- | --- | --- | --- | --- | --- | --- |
| **1-class** | -543.692 | 9 | 1105.384 | 1145.759 | 1117.184 | NA | NA |
| **2-class** | -497.785 | 12 | 1019.570 | 1073.404 | 1035.303 | 0.000 | 0.871 |
| **3-class** | -491.591 | 15 | 1013.181 | 1080.474 | 1032.849 | 0.0128 | 0.871 |
| **4-class** | -485.118 | 18 | 1006.236 | 1086.987 | 1029.836 | 0.0128 | 0.871 |
| **5-class** | -481.743 | 21 | 1005.487 | 1099.696 | 1033.021 | 0.1111 | 0.793 |

**Supplementary Material 4**. Details of the fit indices of the latent growth mixture modelling of Axial Length (AL) for the older cohort, aged 12-22 years. AIC=Akaike Information Criterion, BIC= Bayesian Information Criterion, ssaBIC= sample size-adjusted Bayesian Information Criterion, Boot-LRT= Bootstrap Likelihood Ratio Test. The grey shaded section indicates the best fit for this parameter for this cohort.
